# Identification of potential biomarkers for pathogenesis of Alzheimer’s disease

**DOI:** 10.1186/s41065-021-00187-9

**Published:** 2021-07-05

**Authors:** Huimin Wang, Xiujiang Han, Sheng Gao

**Affiliations:** 1 Department of Neurology, Tianjin Hospital of ITCWM Nankai Hospital, 300100 Tianjin, China; 2 Department of Geriatrics, Tianjin Hospital of ITCWM Nankai Hospital, No.6 Changjiang Road, Nankai, 300100 Tianjin, China

**Keywords:** Alzheimer's disease, Differentially expressed genes, Weighted gene co-expression network analysis, Biomarker

## Abstract

**Background:**

Alzheimer’s disease (AD) is an extremely complicated neurodegenerative disorder, which accounts for almost 80 % of all dementia diagnoses. Due to the limited treatment efficacy, it is imperative for AD patients to take reliable prevention and diagnosis measures. This study aimed to explore potential biomarkers for AD.

**Methods:**

GSE63060 and GSE140829 datasets were downloaded from the Gene Expression Omnibus (GEO) database. The differentially expressed genes (DEG) between AD and control groups in GSE63060 were analyzed using the limma software package. The mRNA expression data in GSE140829 was analyzed using weighted gene co-expression network analysis (WGCNA) function package. Protein functional connections and interactions were analyzed using STRING and key genes were screened based on the degree and Maximal Clique Centrality (MCC) algorithm. Gene Ontology (GO) and Kyoto Encyclopedia of Genes and Genomes (KEGG) enrichment analyses were performed on the key genes.

**Results:**

There were 65 DEGs in GSE63060 dataset between AD patients and healthy controls. In GSE140829 dataset, the turquoise module was related to the pathogenesis of AD, among which, 42 genes were also differentially expressed in GSE63060 dataset. Then 8 genes, RPS17, RPL26, RPS3A, RPS25, EEF1B2, COX7C, HINT1 and SNRPG, were finally screened. Additionally, these 42 genes were significantly enriched in 12 KEGG pathways and 119 GO terms.

**Conclusions:**

In conclusion, RPS17, RPL26, RPS3A, RPS25, EEF1B2, COX7C, HINT1 and SNRPG, were potential biomarkers for pathogenesis of AD, which should be further explored in AD in the future.

**Supplementary Information:**

The online version contains supplementary material available at 10.1186/s41065-021-00187-9.

## Introduction

Alzheimer’s disease (AD) is an extremely complicated neurodegenerative disorder [1], which is usually characterized by progressive decline of abilities to varying degrees, such as memory, language, behavior and so on [2]. Additionally, AD accounts for almost 80 % of all dementia diagnoses and has been the domain cause of dementia [3]. There are more than 45 million people worldwide suffering from AD and the number is estimated to approximate 131 million in next few decades according to a recent report [4]. On the one hand, the increasing number of AD patients is probably caused by the pathological heterogeneity characteristic of AD patients [5]. On the other hand, it has been widely reported that there are two pathologies in AD: β-amyloid plaque deposition and neurofibrillary tangles of hyperphosphorylated tau [6–8], however, no universally acceptable hypothesis could explain the pathogenesis of AD [1, 9]. Accordingly, despite the significant advancements in molecular medicine, the current AD treatments are still not enough to prevent the patients from the irreversible and progressive cognitive decline [1, 10], the negative influence of which is increasing as the aging population in many countries [11]. Collectively, it will be of great significance for all AD patients to take reliable prevention and diagnosis measures in earlier stage. Obviously, potential molecular biomarkers are helpful tools for AD prevention and diagnosis.

At present, it is imperative for those mild cognitive impairment (MCI) and AD patients to receive timely detection, early diagnosis and appropriate management [12]. Lots of researchers devoted to find reliable evidences for the understanding of the molecular pathogenesis of AD, some of which have been transformed into promising treatment approaches [13, 14]. For instance, many biomarkers in cerebrospinal fluid (CSF) has been widely investigated, including amyloid-β (Aβ) [15, 16], total tau levels [17, 18], phosphorylated tau levels [18, 19] and other novel candidate biomarkers. Moreover, it has been demonstrated that more than one biomarker would be more accurate to reveal the probability of AD due to mild cognitive impairment (MCI) [20]. Mean while, some reports revealed that CSF and PET biomarkers were reserved for some certain type of AD in clinical cases, like atypical, rapidly progressive AD and so on [21, 22]. However, due to the invasiveness of CSF collection, this approach is greatly limited in the clinical application [14]. More researches began to explore other biomarkers from easily accessible fluid like plasma and urine. It has been reported that MT1 and several other genes were potential targets for AD therapy, but only weighted gene co-expression network analysis (WGCNA) was included in the study [6]. Another research has suggested that GRIK1 was related to AD stages, via WGCNA analysis [23]. Many previous studies usually focused on one certain gene or one analysis method. Therefore, except for WGCNA, other methods were also used in this research in order to find multiple potential biomarkers associated with the onset of AD, so as to provide more reference information for AD research in the future.

In the present study, two datasets GSE63060 and GSE140829 were downloaded from Gene Expression Omnibus (GEO, https://www.ncbi.nlm.nih.gov/geo/) database, and WGCNA, differential expression analysis and further analysis were integrated in our research. We herein aimed to screen novel potential biomarkers for AD patients through a comprehensive analysis.

## Materials and methods

### Data sources

All mRNA expression data of Alzheimer’s Disease (AD) was downloaded from Gene Expression Omnibus (GEO, https://www.ncbi.nlm.nih.gov/geo/) database. The GSE63060 dataset [24] included 145 blood samples from AD patients and 104 blood samples from healthy controls. The mRNA expression data of this dataset was detected using the Illumina HumanHT-12 V3.0 expression beadchip platform. Another dataset GSE140829 included 204 blood samples from AD patients and 249 blood samples from healthy controls. The mRNA expression data of GSE140829 dataset was detected using HumanHT-12 v4 Expression BeadChip platform. The clinical characteristics of the samples in these two datasets were shown in Table S1.

### Differential expression analysis

The differentially expressed genes (DEG) between AD and control groups were analyzed using the limma software package [25] in R language. The |log_2_FC|>0.5 and adjusted *P* value < 0.05 (P_FDR_<0.05) after multiple testing by Benjamini and Hochberg (BH) method were used as the standards to screen the DEGs.

### Weighted gene co-expression network analysis

The mRNA expression data in GSE140829 was analyzed using WGCNA function package [26] in the R language. Via this method, all genes would be hierarchically clustered according to the gene expression value, then modules were identified using dynamic tree cutting method and those genes with higher similarity would be classified into the same module. Subsequently, the Module Eigengene (ME) value of each module was calculated. Additionally, the correlation coefficient between the ME value and certain phenotypes (such as type of disease, gender, age and so on) was calculated. When *p* value was less than 0.05, the larger the correlation coefficient, the closer relationship between this module and the phenotype.

### Functional enrichment analysis

Gene ontology (GO) and Kyoto Encyclopedia of Genes and Genomes (KEGG) pathway enrichment analyses were performed using clusterProfiler [27] package in R language. GO enrichment analysis included Biological Process (BP), Cellular Component (CC) and Molecular Function (MF) terms. The GO terms and KEGG pathways with *P*. adjust < 0.05 were considered to be significantly enriched.

### Protein-protein interaction (PPI) network analysis

STRING is a database used for protein functional connection and protein-protein interaction analyses. Here, protein functional connections and interactions were analyzed using STRING (https://string-db.org/,version 11.0) [28]. The interaction pairs with confidence score ≥ 0.4 were retained. The PPI network was then visualized using Cytoscape (version 3.7.2) (https://cytoscape.org/) [29]. The key genes in PPI network were screened based on the degree of nodes and Maximal Clique Centrality (MCC) algorithm, using cytoHubba plug-in of Cytoscape software.

## Results

### Differentially expressed genes

**Fig. 1:**
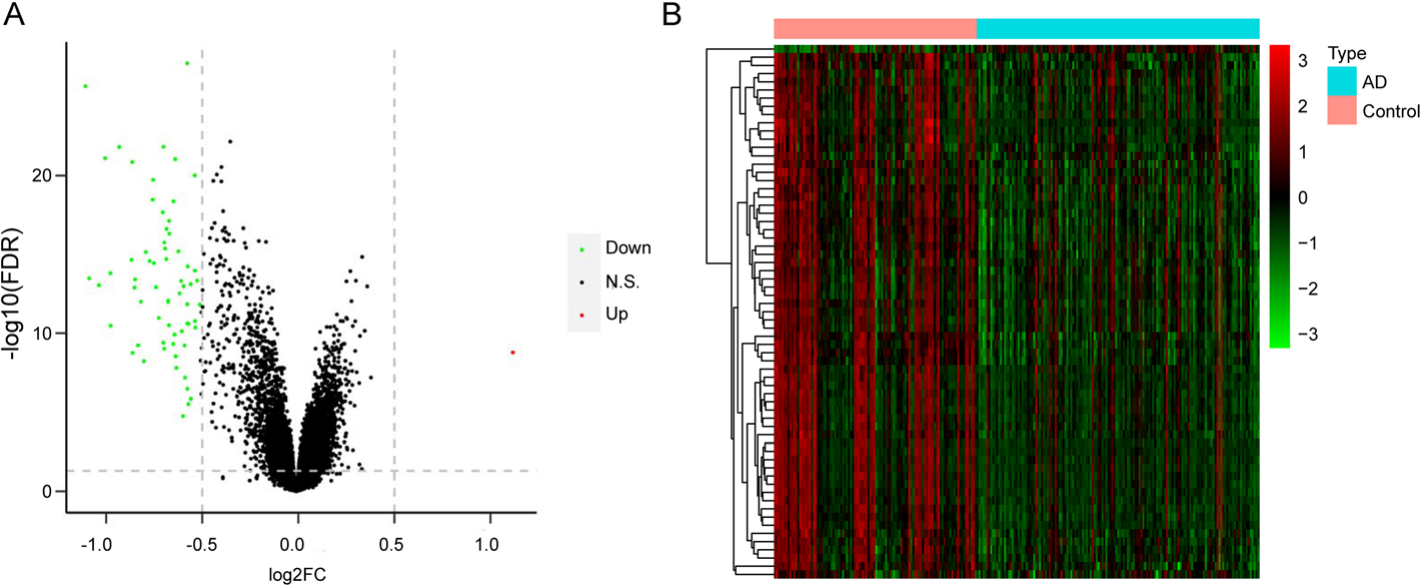
Differentially expressed genes.** A** Volcano map of differentially expressed genes in GSE63060 dataset. Horizontal axis: the log_2_FC value; vertical axis: -log10 (FDR). Red: up-regulation; green: down-regulation; black: non-significant difference. **B** Expression level heat map of differentially expressed genes in GSE63060. Horizontal axis: genes; vertical axis: samples. Red: high expression; green: low expression

Based on all mRNA expression data in GSE63060 dataset, the DEGs between AD and healthy control groups were identified. Between AD patients and healthy controls, there were 65 DEGs, including 1 up-regulated gene and 64 down-regulated genes (Fig. 1A). The expression levels of these DEGs were significantly different between the two groups (Fig. 1B).

### Potential genes related to AD occurrence identified via WGCNA analysis

**Fig. 2:**
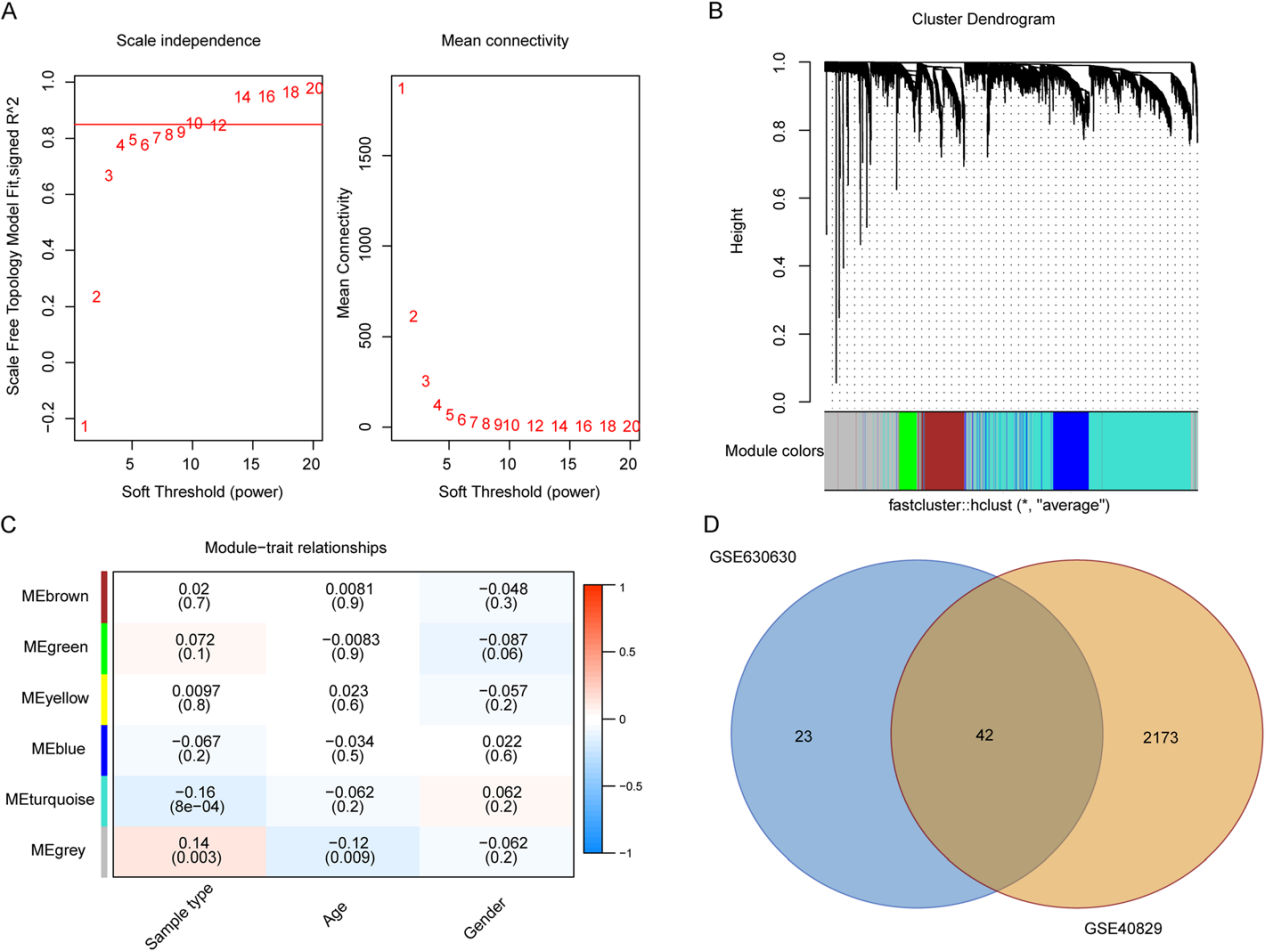
Results of WGCNA.** A** Schematic diagram of soft threshold screening. Red line represented the square of the correlation coefficient, 0.85, and the first point above red line was the soft threshold β = 10. **B** Schematic diagram of gene module clustering. Each color represented a module, and the gray module included the genes that could not be clustered into any module. **C** Heat map of the correlation between gene module and phenotype. Red: positive correlation; blue: negative correlation. The darker the color, the greater the correlation. **D** Venn diagram of differentially expressed genes in GSE63060 dataset and genes screened via WGCNA in GSE140829 dataset

WGCNA analysis was performed on the samples in GSE140829 dataset. The results showed that the gene co-expression network conformed to unsigned network. The higher the square of the correlation coefficient, the closer the network was to the distribution of unsigned network. The square of the correlation coefficient, 0.85, was taken as the standard to select the soft threshold (β = 10, Fig. 2A).

Then the genes were clustered based on average-linkage hierarchical clustering method. According to the standard of dynamic tree cutting, 100 was set as the minimum number of genes in each gene network module. Subsequently, module eigengene (ME) value of each module was calculated in turn and cluster analysis was performed on the modules. The close modules were merged into a new one. With the height of 0.25, 6 modules were finally obtained (Fig. 2B). The gray module contained all genes that could not be clustered in other modules.

According to the ME values of the obtained modules, the correlations between these modules and the phenotypes were calculated. The results suggested that the correlation between the turquoise module and sample type was the largest (correlation coefficient was − 0.16, *P* = 8e-4) (Fig. 2C), which indicated that the genes in turquoise module (*n* = 2215) obtained via WGCNA were potentially related to the pathogenesis of AD. Moreover, 42 of the 2215 genes in turquoise module were also differentially expressed in GSE63060 dataset (both were up-regulated in AD), which implied that these 42 genes were potentially more associated with the pathogenesis of AD (Fig. 2D).

### Significantly enriched GO terms and KEGG pathways

**Fig. 3:**
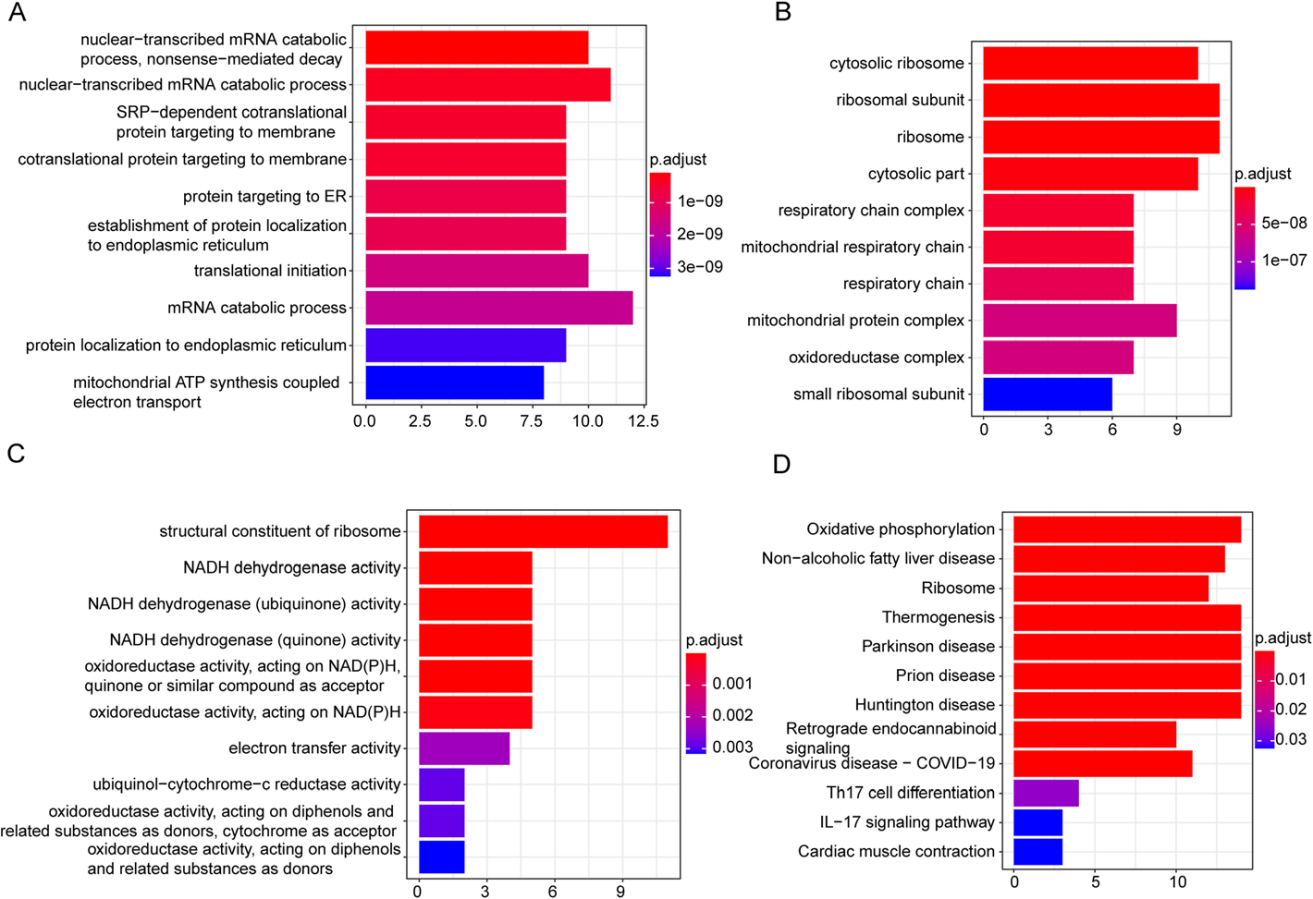
Significantly enriched GO terms and KEGG pathways.**A** The top 10 significantly enriched Biological Process terms. **B** The top 10 significantly enriched Cellular Component terms. **C** The top 10 significantly enriched Molecular Function terms. **D** The 12 significantly enriched KEGG pathways. Horizontal axis: the number of enriched genes; vertical axis: the corresponding biological process; different *p*.adjust values were represented by altered colors

In order to obtain more function information of the 42 shared genes, GO and KEGG pathway enrichment analyses were performed. GO terms were significantly enriched, including 51 Biological Process terms, 52 Cellular Component terms and 16 Molecular Function terms. The top 10 significantly enriched Biological Process terms (Fig. 3A), Cellular Component terms (Fig. 3B) and Molecular Function terms (Fig. 3C) were displayed in Fig. 3A-C. There were 12 KEGG pathways that were significantly enriched (Fig. 3D). The detailed results of GO and KEGG pathway enrichment analyses were shown in Table S2.

### PPI network construction and key genes screening

**Fig. 4:**
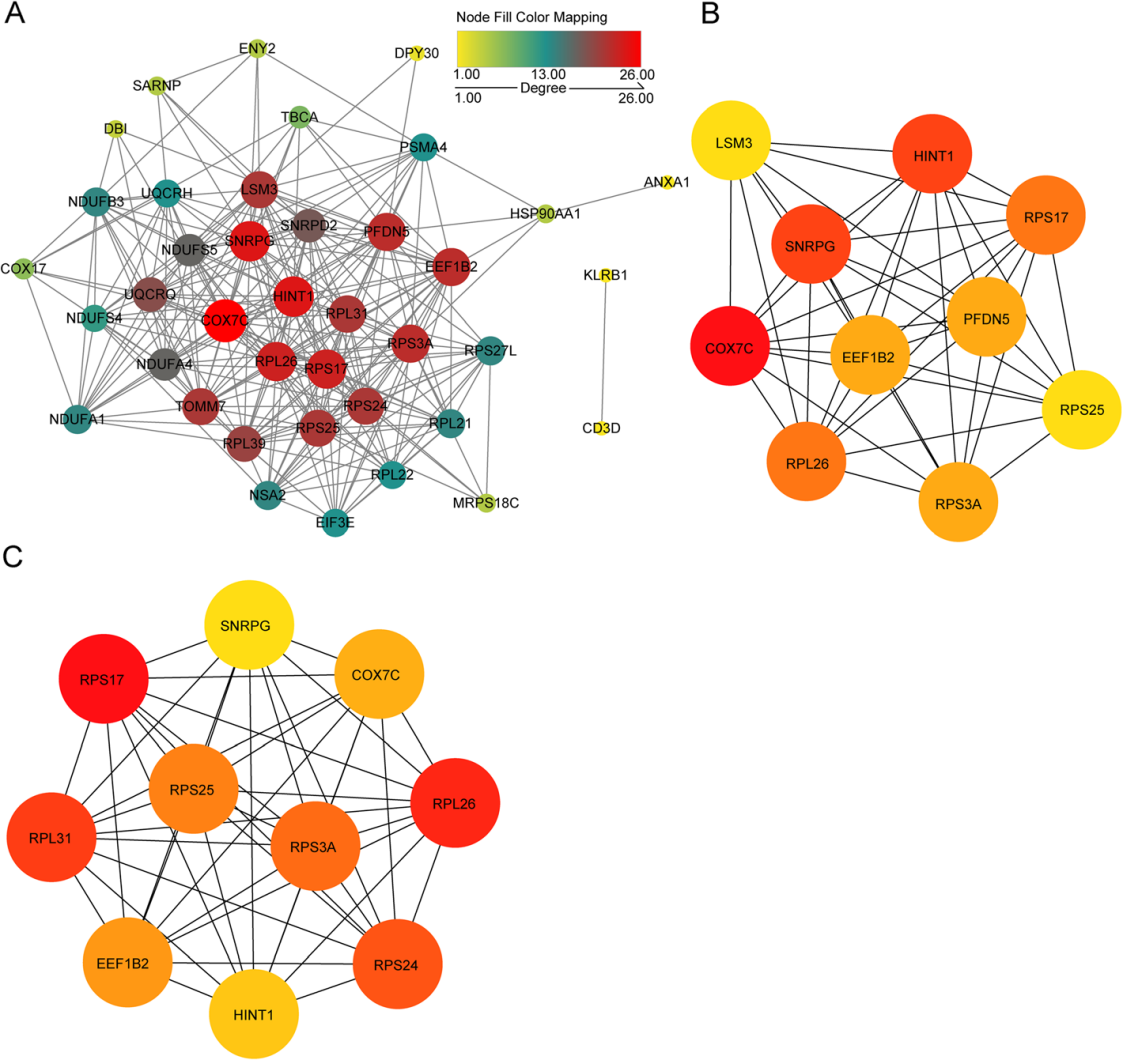
PPI network construction and key genes screening.** A** Diagram of protein-protein interaction network. Each dot represented a node. The more line segments connected to the node, the greater the degree of this node. The degree of node was reflected by its size and color. The larger the node, the deeper color from yellow to red, the greater the degree. **B** Network diagram of the top 10 genes screened according to degree. The darker color from yellow to red represented greater degree. **C** The network diagram of the top 10 genes screened via MCC algorithm. The darker color from yellow to red represented greater score

The 42 genes were chosen to construct the PPI network. The interaction pairs with confidence score ≥ 0.4 were visualized using Cytoscape software (Fig. 4A). Among the 42 genes, there were 39 interacting genes. Among them, COX7C had the highest node degree of 26, and the lowest node degree was 13. The top 10 genes screened according to the degree of the node were displayed in Fig. 4B, using cytoHubba plug-in of Cytoscape software. The top 10 genes screened according to the MCC algorithm were shown in Fig. 4C, among which, 8 genes were overlapped with the top 10 genes screened by the degree. The results suggested that these 8 genes, including RPS17, RPL26, RPS3A, RPS25, EEF1B2, COX7C, HINT1 and SNRPG, were more crucial to the pathogenesis of AD. Detailed degree and MCC algorithm score of these 8 genes were displayed in Table 1.

**Table 1:** The degree and MCC score of the selected 8 genes

Gene	Degree	MCC score
RPS17	23	1.05E + 09
RPL26	23	1.05E + 09
RPS3A	22	1.04E + 09
RPS25	21	1.04E + 09
EEF1B2	22	1.01E + 09
COX7C	26	9.68E + 08
HINT1	24	9.64E + 08
SNRPG	24	9.63E + 08

Additionally, as differentiating AD from other diseases with similar symptoms is of great clinical significance, we also established a logistic regression model based on the selected 8 genes to further evaluate our biomarkers in distinguishing AD from mild cognitive impairment (MCI), another type of dementia disorder. As shown in Figure S1, the area under curve (AUC) value of the established model was 0.6021, indicating that the model based on the selected genes had potential value in differentiating AD from MCI. However, this is only a preliminary research on the utility of the selected genes, and further optimization is needed for their pervasive application in this regard.

## Discussion

In this research, via an integrated analysis including WGCNA, differential expression analysis and other further analyses of the data downloaded from GEO database, we have explored the possible biomarkers for AD patients. There were 65 DEGs between AD patients and healthy controls in GSE63060 dataset. Subsequently, via WGCNA, the turquoise module was found to be related to the pathogenesis of AD. Finally, 8 genes, including RPS17, RPL26, RPS3A, RPS25, EEF1B2, COX7C, HINT1 and SNRPG, were evidenced to be crucial to the pathogenesis of AD.

Two datasets, GSE63060 and GSE140829, were included in our research. Firstly, in GSE63060 dataset, 65 genes were found to be differentially expressed between AD patients and healthy controls, including 1 up-regulated gene and 64 down-regulated genes. Then, WGCNA analysis was performed on the samples in GSE140829 dataset, through which, 6 co-expression modules were obtained and the turquoise module was related to the pathogenesis of AD. Among all 2215 genes in turquoise module, 42 genes were also differentially expressed in GSE63060 dataset. As far as we know, most of the previous studies only used WGCNA to find AD related genes [6, 30], but we have integrated the results of DEGs and WGCNA in order to obtain a more reliable basis before further analysis. Based on the PPI network, 8 genes among all 42 genes were finally screened according to the degree and MCC algorithm, which included RPS17, RPL26, RPS3A, RPS25, EEF1B2, COX7C, HINT1 and SNRPG. Some direct or indirect evidence was found to support most of the 8 genes. Regarding RPL26, Mastroeni at al. suggested that it was a marker of ribosome in neurons and could be used in methylation related studies in AD neurons in the future [31]. And a recent research demonstrated that RPS3A was not only major pathogenic gene of MCI, but bridge gene; SNRPG was not only major pathogenic gene of MCI and AD, but also bridge gene [32]. Our results were consistent with the previous researches to some extent. It has been suggested that RPS25 might be a potential therapeutic target for C9orf72-related neurodegenerative diseases caused by nucleotide repeat expansions [33], which could support our findings indirectly. A study documented that AEEF1B2 belonged to the eukaryotic elongation translational machinery and AEEF1B2 variants in the translational machinery were associated with several neurodevelopmental disorders [34]. It has been reported that the expression of COX7C was decreased in total homogenates of the entorhinal cortex in AD stages V-VI [35]. However, RPS17 and HINT1 have not been studied in AD, which should be investigated in the future. Collectively, these 8 genes were potential biomarkers for pathogenesis of AD and they could be further explored in AD researches.

In addition, GO and KEGG pathway enrichment analyses were performed on the 42 shared genes in order to obtain more function information of them. GO terms were significantly enriched, including 51 Biological Process terms, 52 Cellular Component terms and 16 Molecular Function terms. The 42 genes were significantly enriched in 12 KEGG pathways, including Oxidative phosphorylation, Non-alcoholic fatty liver disease (NAFLD), Parkinson disease and several other pathways. Some of the KEGG pathways were related to the previous studies to some extent and they partially provided new ideas for future researches. For example, several studies have demonstrated the complicated roles of oxidative stress in the pathogenesis and progression of AD [36–38], which could be linked to the oxidative phosphorylation pathway in further studies. Regarding Non-alcoholic fatty liver disease (NAFLD) pathway, it has been reported that NAFLD-induced chronic inflammation induced neurodegeneration diseases in wild-type mice [39], which could also be correlated with inflammation in AD as inflammation had been evidenced to be associated with the pathogenesis of AD [40, 41]. In addition, some genes were significantly enriched in Parkinson disease pathway, which indicated that Parkinson disease and AD might be explored together, as age-related neurodegeneration diseases [42, 43]. Collectively, these pathways have provided more information for the further exploration of AD in the near future and we would also spare no effort to continue the researches on AD.

## Conclusions

In conclusion, via integrating the results of WGCNA and DEGs, 8 genes, including RPS17, RPL26, RPS3A, RPS25, EEF1B2, COX7C, HINT1 and SNRPG, were further screened and evidenced to be associated with the occurrence of AD. These genes were significantly enriched in Oxidative phosphorylation, NAFLD and some other pathways. Our findings will provide more information for this complicated neurodegenerative disorder.

## Supplementary Information

**Additional file 1: Figure S1. **Establishment of the logistic regression model based on the selected 8 genes. (A) Residuals vs leverage plot to detect the influential point. The red dashed line indicated the COOK distance. The point with a COOK distance greater than 0.05 was considered as an influential point, which could affect the reliability of the model. It was shown that there were no influential points for our model. (B) Component plus residual plots of the selected 8 genes. The obvious linear relationship between the horizontal axis and vertical axis indicated that the independent variable could be included in the model. (C) The ROC curve. The horizontal axis denoted the false positive rate, and the vertical axis denoted the true positive rate.

**Additional file 2.** The clinical characteristics of samples in GSE63060 and GSE140829 datasets.

**Additional file 3: Table S2.** Significantly enriched GO entries and KEGG pathways.

## Data Availability

The data that support the findings of this study are available in (GEO) at (https://www.ncbi.nlm.nih.gov/geo/).

## Abbreviations

AD: Alzheimer's disease
GEO: Gene Expression Omnibus
DEG: Differentially expressed genes
WGCNA: Weighted gene co-expression network analysis
MCC: Maximal Clique Centrality
GO: Gene Ontology
KEGG: Kyoto Encyclopedia of Genes and Genomes
MCI: Mild cognitive impairment
CSF: Cerebrospinal fluid
ME: Module Eigengene
BP: Biological Process
CC: Cellular Component
MF: Molecular Function
PPI: Protein-protein interaction
AUC: Area under curve
NAFLD: Non-alcoholic fatty liver disease
