# Supplementary figures and images for: Identification of potential biomarkers for pathogenesis of Alzheimer’s disease
Source: Hereditas. 2021 Jul 5;158:23. doi: 10.1186/s41065-021-00187-9 (PMC8259215; doi:10.1186/s41065-021-00187-9)

A

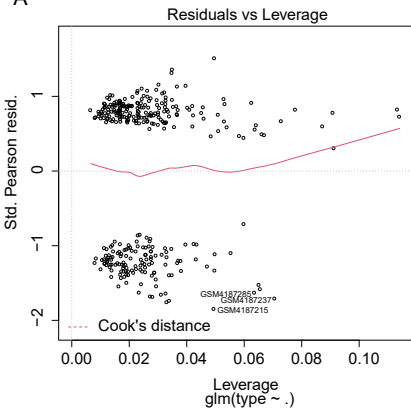

C

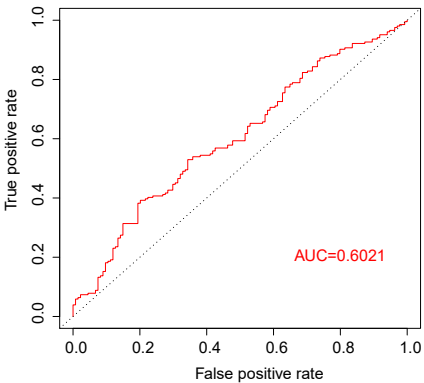

B

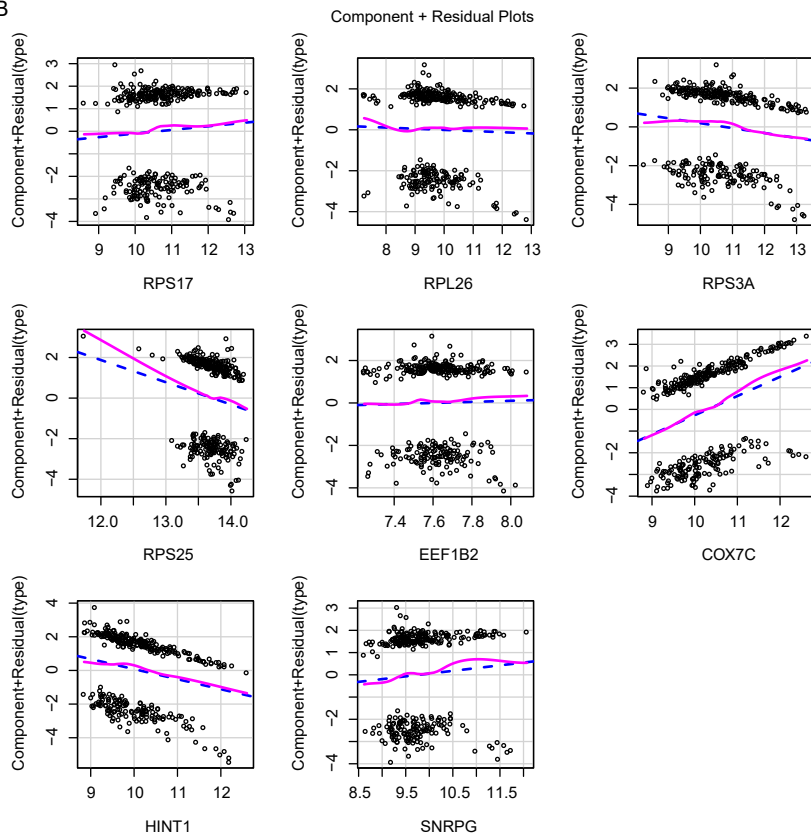

Supplement: Supplementary file 1 — Additional file 1: Figure S1. Establishment of the logistic regression model based on the selected 8 genes. (A) Residuals vs leverage plot to detect the influential point. The red dashed line indicated the COOK distance. The point with a COOK distance greater than 0.05 was considered as an influential point, which could affect the reliability of the model. It was shown that there were no influential points for our model. (B) Component plus residual plots of the selected 8 genes. The obvious linear relationship between the horizontal axis and vertical axis indicated that the independent variable could be included in the model. (C) The ROC curve. The horizontal axis denoted the false positive rate, and the vertical axis denoted the true positive rate. [file 41065_2021_187_MOESM1_ESM.pdf]
